# Accuracy of Implant Positioning in Total Hip Arthroplasty via a Supine Direct Anterior Approach Using Fluoroscopy

**DOI:** 10.1155/aort/8892577

**Published:** 2025-03-12

**Authors:** Keijiro Kanno, Shigeo Hagiwara, Yuki Shiko, Yuya Kawarai, Junichi Nakamura, Seiji Ohtori

**Affiliations:** ^1^Department of Orthopaedics Surgery, Graduate School of Medicine, Chiba University, 1-8-1 Inohana, Chuo-ku, Chiba 260-8670, Japan; ^2^Biostatistics Section, Clinical Research Center, Chiba University Hospital, 1-8-1 Inohana, Chuo-ku, Chiba 260-8670, Japan

**Keywords:** direct anterior approach, implant position, propensity score matching, total hip arthroplasty

## Abstract

**Background:** Adequate implantation is important to avoid complications following total hip arthroplasty (THA). This study aimed to evaluate the accuracy and precision of implant placement in the direct anterior approach (DAA) using fluoroscopy in comparison with the anterolateral approach in lateral decubitus position (OCM) using a single implant.

**Methods:** We retrospectively compared propensity score-matched THAs in DAA with fluoroscopy and in OCM. The achievement ratio of the Lewinnek cup safe zone, absolute difference in alignment, and positioning from preoperative planning was evaluated and compared between each approach.

**Results:** 33 patients in both groups were eligible for this study. Significantly more cups were inside the safe zone in the DAA group than in the OCM group (33/33 vs. 25/33, *p*=0.0048). No significant differences were found between the DAA group and OCM group regarding the discrepancy from the target cup inclination, anteversion, and three-dimensional positioning. No significant difference was noted in stem alignment; however, the equality of coronal alignment variances was smaller in the DAA group (*p*=0.0047). No significant differences were found in the clinical score and complication rate.

**Conclusion:** The DAA using fluoroscopy may provide more accuracy for cup placement and precision for stem placement than OCM.

## 1. Introduction

Accurate cup and stem positioning in total hip arthroplasty (THA) is considered essential to prevent postoperative complications such as impingement, dislocation, and polyethylene wear [1–3]. Recently, various devices such as computer navigation systems and robotic-assisted surgery have been used to ensure the accuracy and precision of cup and stem positioning, and their results were reported to be good [3–5]. However, the surgery using these devices is very expensive, and available facilities are limited [6, 7].

Judet et al. reported the Hueter anterior approach for the first time in 1950 in relation to THA [8]. Since the patient undergoing THA via the direct anterior approach (DAA-THA) is placed supine on the operating table, the anterior approach permits the use of fluoroscopic image intensification, allowing intraoperative assessment and correction of component positioning, which may permit a more accurate final-component position [9]. An increasing number of surgeons demonstrated interest in the DAA for its minimal invasiveness and the visuality of the component [10]. The OCM approach, which is the anterolateral (AL) approach in the lateral decubitus position, is also introduced for minimally invasive intermuscular invasion [11]. However, it is difficult to perform surgery with fluoroscopy because of the lateral decubitus position. The use of fluoroscopy during surgery improves cup placement in both DAA and posterior approach [12]. However, the advantage of DAA-THA using fluoroscopy compared with THA with the OCM approach is unclear. Thus, this study aimed to evaluate the accuracy and precision of implant placement in DAA using fluoroscopy in comparison with the AL approach in the lateral decubitus position.

## 2. Materials and Methods

**Figure 1:**
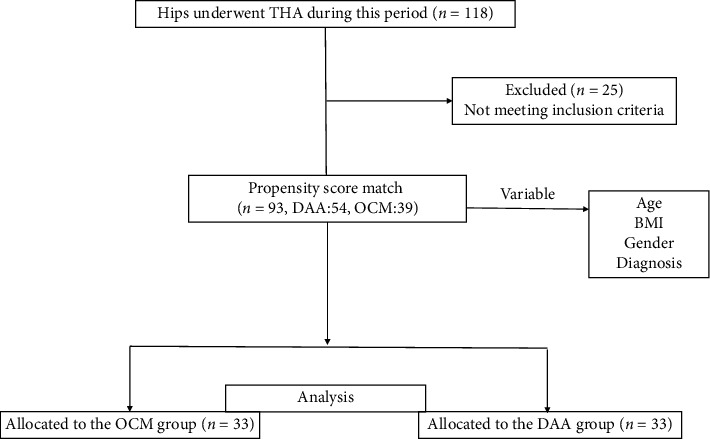
Study selection flow diagram.

This retrospective cohort study included consecutive patients who underwent DAA-THA in the supine position and OCM in the lateral decubitus position in our institute with a single implant between July 2011 and January 2016. The inclusion criteria was primary THA with SL-Plus MIA and R3 cup (Smith and Nephew Orthopaedics, Memphis, Tennessee) using three-dimensional (3D) preoperative planning software ZedHip (Lexi, Tokyo, Japan) before THA [10]. A total of 118 hips underwent primary THA with the implant combination during this period. Patients who had undergone hip surgery including THA and had osteotomy and osteosynthesis and missing outcome data were excluded. After the implementation of the exclusion criteria, 93 hips were selected. The DAA and OCM groups were matched in a 1:1 ratio using propensity scores (PSs) to adjust for age, body mass index (BMI), gender, diagnosis (osteoarthritis or osteonecrosis of the femoral head) (Figure 1).

The study protocol was in compliance with the Helsinki Declaration and approved by the institutional review board (Graduate School of Medicine, Chiba University #2986). All patients gave written informed consent for the use of information.

### 2.1. Surgical Procedure

All THA procedures were supervised by two senior surgeons (NJ or KS). Preoperative computed tomography (CT) scans of the femurs of all patients were acquired. ZedHip software was used for preoperative planning of THA. Digital images in medicine data for each patient were transferred to ZedHip, which then created a 3D model of the acetabulum and femoral head. Preoperative planning was performed to restore the hip center of rotation, femoral offset, and leg lengths, as previously described [13].

**Figure 2:**
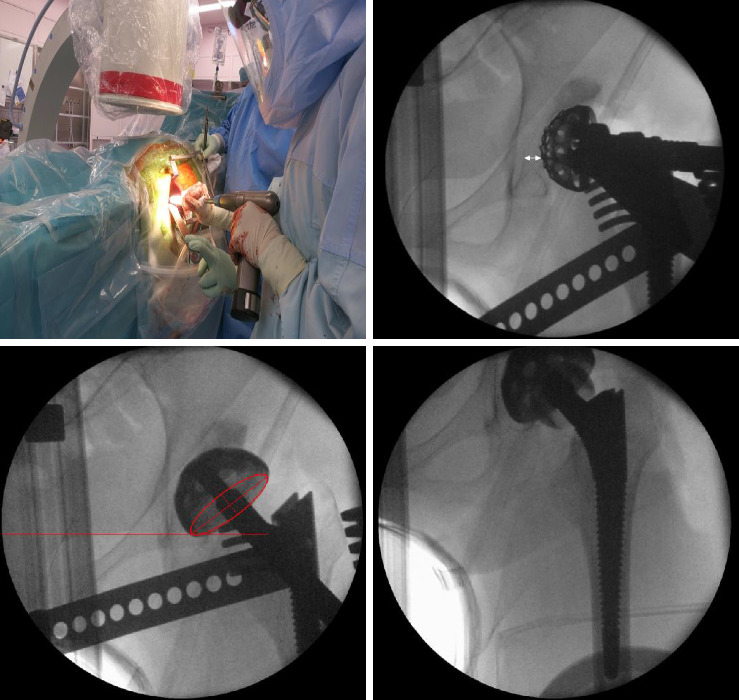
Direct anterior approach using fluoroscopic image allows assessment of reaming and placement of the acetabular cup. The depth of acetabular was evaluated before reaming (white allow). Radiographic inclination and anteversion of the cup were adjusted under fluoroscopic guide (red line). Stem alignment was checked using final broach.

In the DAA group, THA was performed in the supine position, and fluoroscopy was used during reaming and final implant placement to check the cup position angle. Stem alignment was also checked under fluoroscopic visualization. All exposures were performed within 20 s. In the OCM group, the AL approach was used in the lateral decubitus position, and during surgery, control X-ray imaging was performed once before the final implant placement. Cup alignment was evaluated regarding inclination and anteversion according to the method of Lewinneck et al. (Figure 2) [14].

### 2.2. Postoperative CT Measurement of the Component and Clinical Outcome

Using 3D software ZedHip, the postoperative component position can be also compared with the position planned preoperatively. Using skeletal reference points, the coordinates of the acetabular and femoral sides can be determined. Each coordinate was also adapted for postoperative implant positioning and alignment evaluation. The *x*-, *y*-, and *z*-axes were defined as previously reported [15]. These are consistent with the convention previously established by Murray et al. [16]. Radiographic inclination (RI) and radiographic anteversion (RA) were evaluated for the acetabular component alignment. The varus–valgus angle and flexion–extension angles were evaluated for the femoral component alignment. The flexion–extension angle is the angle between the proximal bone axis and the femoral component on the sagittal plane. For the 3D positioning of the acetabular components, the distance between the postoperative implant position and the preoperatively planned position was measured. The software used the preoperative plan as a reference point for the coordinates. 3D distance axes were defined according to acetabular component coordinate systems. The main outcome measures were the absolute deviation of the postoperatively measured angle from the target position and the number of cups inside the Lewinnek safe zone (30°–50° of abduction and 5°–25° of anteversion) [14]. As clinical results, complications (such as dislocation, infection, and fracture) and postoperative The Japanese Orthopaedic Association (JOA) score were also examined in both groups.

### 2.3. Statistical Analysis

Continuous variables were expressed as the mean ± standard deviation. Categorical data were presented as absolute numbers and percentages. Continuous variables were compared using Student's *t*-test. Categorical variables were compared using Pearson's chi-square test or Fisher's exact test. PSs were calculated for each participant using multivariate logistic regression based on the four independent variables, namely, age, BMI, gender, and diagnosis. We conducted a 1:1 PS matching analysis without replacement (greedy matching algorithm), with a caliper width equal to 0.2 of the standard deviation of the logit of the PS. To examine the balance of covariate distribution between the OCM and DAA groups, we calculated the standardized difference.

For each outcome, the mean and equality of variance between groups were assessed by Student's *t*-test and *F* test in the PS-matched groups. The proportion of cups inside the Lewinnek safe zone between groups was compared using Fisher's exact test. A *p* value < 0.05 was considered significant, and all statistical analyses were performed using the SAS statistical software package version 9.4 (SAS Institute, Cary, USA).

## 3. Results

The demographic data of the patients are shown in Table 1. After PS matching, 33 patients in both groups were eligible for this study. No significant differences were found regarding gender, age, BMI, and preoperative diagnosis between the two groups. No significant difference was found in the postoperative JOA scores between the two groups (*p*=0.2697, Table 1).

**Figure 3:**
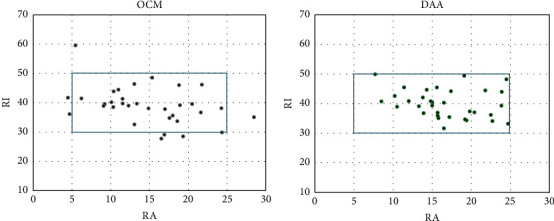
Scatter plot of the distribution of the cup placement angles in the anterolateral approach (OCM) and direct anterior approach (DAA) groups. RA, radiographic anteversion; RI, radiographic inclination.

The postoperative RI values were 39.9 ± 4.6 in the DAA group and 39.0 ± 6.2 in the OCM group. The postoperative RA measurements were 16.7 ± 4.5 in the DAA group and 14.8 ± 5.8 in the OCM group (Figure 3).

**Figure 4:**
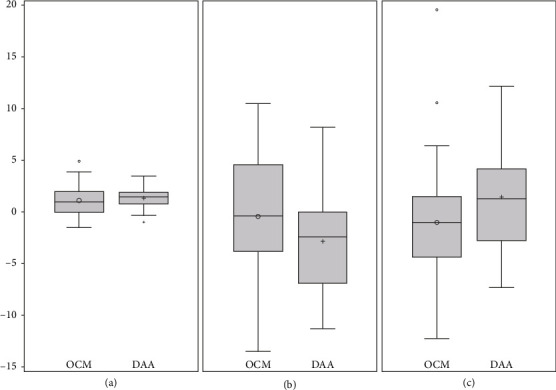
Box plots of discrepancy from target. (a) Stem varus/valgus, (b) cup anteversion, and (c) cup inclination angles in the anterolateral approach (OCM) and direct anterior approach (DAA) groups.

No significant differences were noted between the OCM group and DAA group regarding the discrepancy from the target to the postoperatively measured angles in the RI and RA (Figure 4). The F test showed no significant difference in the equality of variance in the RI between each group (Table 2). In addition, no significant differences were noted in the 3D distance in the *x*-, *y*-, and *z*-axis (Table 2).

The postoperative stem varus–valgus angles were 1.52° ± 1.16° in the OCM group and 1.43° ± 0.79° in the DAA group (*p*=0.712, Figure 4). As per the F test for the equality of variances, these differences were significant (*p*=0.0047, Table 3). The postoperative stem flexion/extension angles were 3.05° ± 1.27° in the OCM group and 3.24° ± 1.26° in the DAA group (*p*=0.554). As per the F test for the equality of variances, these differences were not significant (*p*=0.9693, Table 3).

The number of cups inside the Lewinnek safe zone was 33/33 (100%) in the DAA group and 25/33 (78.7%) in the OCM group. Significantly more cups were inside the Lewinnek safe zone in the DAA group than in the OCM group (*p*=0.0048, Figure 3). In the DAA group, one dislocation occurred. In the OCM group, two dislocations and one infection occurred (Table 1).

## 4. Discussion

This study investigated the accuracy and precision of implant placement in THA using different approaches. Although no differences were found in the cup alignment and position from the preoperative planning, the cups were likely to be inside the safe zone in the DAA group. The stems were significantly in the neutral position in the DAA group. The short-term complication rate was the same in both groups.

Implant positioning and alignment are important because inadequate cup placement can lead to an increased incidence of dislocation, increased polyethylene wear, limited range of motion, and poor THA outcomes [2, 14, 17–19]. THA via a lateral position has been the worldwide standard procedure, and position fixation device is used, in recent years, as reported in the Australian registry [20]. Essentially, pelvis fixation in the lateral position has been reported to show greater variance among facilities and even among individuals than pelvis fixation in the supine position because of sagittal tilt, rotation, and lateral tilt [21, 22]. Variance in the sagittal and coronal tilt of the pelvis during lateral position THA is known to affect the precision of the anteversion for cup placement.

The introduction of a navigation system is thought to solve the variance in implant alignment and positioning. CT-based navigation achieved a mean absolute error of < 2° for cup inclination and anteversion [23]. However, the navigation system can be limited by cost, increased operative time for the calibration, extra incision for the tracker, and technical problems [19]. Recently, robot-assisted THA has garnered considerable interest to improve implant placement and reduce complications [24]. Barger et al. reported that robot-assisted surgery has high initial costs but may provide sufficient benefit [25]. Many reports have shown superior cup placement in robot-assisted THA, such as navigation systems, compared with conventional methods [3, 26–28]. Regarding clinical results, robot-assisted surgery had a low dislocation rate [24], whereas Illgen et al. reported that although the dislocation rate decreased, no significant difference was noted [26]. Fluoroscopy is a normal device for most of the institutes and does not require extra installation cost.

Barrett et al. [29] reported the improvement of cup anteversion with the DAA compared with the posterior approach. With the use of fluoroscopy, Rathod et al. demonstrated that the DAA can result in more accurate THA-component positioning compared with an unguided posterior approach [30]. Matta et al. [31], who described their results with the DAA using fluoroscopy, reported that cup position is more reliable with this technique, as target cup positions were achieved in most patients. Using fluoroscopy, the surgeon can modify the cup alignment during surgery. In this study, no significant difference was found in the average deviation of the cup RI and RA from the target position in the DAA and OCM groups. However, the DAA was superior in accuracy; as a result, the achievement rate of the safe zone was 100%. In this study, THA was performed after 3D templating in all cases. 3D templating allows evaluation of the diameter of the acetabulum, position of the osteophyte, and depth of the double floor, which may lead to the accurate positioning of the cup in both approaches.

Most of the studies using navigation and robotic-assisted THA evaluated the placement of the cup. However, stem alignment can also affect impingement and dislocation [32]. A recent comparison study of stem alignment between supine and lateral positions reported more frequent flexed stem insertion with the supine position and claimed that the traction table may solve the flexed implant insertion owing to the ideal leg position [33]. Although our study showed no difference in the coronal and sagittal alignment between each group, the equality of variances in the DAA group was smaller than that in the OCM group. DAA using traction table plus fluoroscopy enable modification of coronal stem alignment.

This study has several limitations. First, we used only one implant combination. It is unclear whether the results of this study can be generalizable for other implants. However, the position and alignment can be evaluated using fluoroscopy because most of the cementless cups are spherical. Second, this is a retrospective nonrandomized comparative study with a small sample size. However, we conducted PS matching analysis to minimize bias. Finally, even if PS methods can reduce bias in causal estimates due to the observed differences between groups, they are still subject to biases in unobserved differences.

In Conclusion, our PS matching analysis suggested that the DAA using fluoroscopy provided more accuracy for cup placement and precision for stem placement without complication than the AL approach in the lateral position. However, further study is needed to determine the long-term clinical advantage for the DAA.

## Figures and Tables

**Table 1:** Patient characteristics (before and after propensity score matching).

	Before PS matching				After PS matching			
	OCM (*n* = 39)	DAA (*n* = 54)	*p* value	Standardized difference	OCM (*n* = 33)	DAA (*n* = 33)	*p* value	Standardized difference
Gender, female/male, *n*	11/28	13/41	0.653	0.094	10/23	11/22	0.792	0.069
Age (y), mean (SD)	60.8 (8.75)	58.4 (11.79)	0.293	0.228	60.5 (9.10)	59.8 (12.25)	0.785	0.070
BMI (kg/m^2^), mean (SD)	31.3 (8.49)	28.5 (4.84)	0.052	0.397	29.5 (6.70)	29.0 (5.58)	0.738	0.074
Preoperative diagnosis, *n* (%)								
OA	27 (69.2)	35 (64.8)	0.656	0.094	22 (66.7)	20 (60.6)	0.609	0.129
ON	12 (30.8)	19 (35.2)			11 (33.3)	13 (39.4)		
Clinical outcome								
JOA score, mean (SD)					88.7 (9.27)	91.1 (8.63)	0.270	—
Dislocation, *n* (%)					2 (6.1)	1 (3.0)	0.555	—
Infection, *n* (%)					1 (3.0)	0 (0)	0.314	—
Fracture, *n* (%)					0 (0)	0 (0)	—	—

*Note:* Data are shown as mean ± standard deviation, or number (%). ON, osteonecrosis of the femoral head.

Abbreviations: BMI, body mass index; OA, osteoarthritis; SD, standardized difference.

**Table 2:** Cup discrepancy from the target angle and position in the *x*-, *y*-, and *z*-axes (absolute value).

	OCM	DAA	*p* value
Discrepancy from the target angle (absolute value)			
Inclination (°)	4.79 ± 4.62	3.89 ± 2.98	0.3542
Anteversion (°)	4.87 ± 3.33	4.78 ± 3.32	0.9064
Distance from the target cup position (mm)–*X* axis-	4.26 ± 2.96	2.33 ± 1.59	0.1885
Distance from the target cup position (mm)–*Y* axis-	2.30 ± 1.62	1.65 ± 1.16	0.1832
Distance from the target cup position (mm)–*Z* axis-	2.58 ± 2.36	2.34 ± 1.24	0.879

*Note:* OCM, anterolateral approach in lateral decubitus position.

Abbreviation: DAA, direct anterior approach.

**Table 3:** Stem discrepancy from the target angle (absolute value).

	OCM	DAA	*p* value (T test)	*p* value (F test)
Discrepancy from the target angle (absolute value)				
Varus/valgus (°)	1.52 ± 1.16	1.43 ± 0.79	0.712	0.0047
Flexion/extension (°)	3.05 ± 1.27	3.24 ± 1.26	0.554	0.9693

*Note:* OCM, anterolateral approach in lateral decubitus position.

Abbreviation: DAA, direct anterior approach.

## Data Availability

The datasets generated during the current study are available from the corresponding author on reasonable request.
